# Systematic review and randomized controlled trial meta-analysis of the effects of physical activity interventions and their components on repetitive stereotyped behaviors in patients with autism spectrum disorder

**DOI:** 10.3389/fpsyg.2025.1579345

**Published:** 2025-05-23

**Authors:** Juanjuan Yang, Ruxin Li

**Affiliations:** ^1^School of Physical Education and Health, Shanghai Lixin University of Accounting and Finance, Shanghai, China; ^2^Sports Institute, Hunan University of Finance and Economics, Changsha, Hunan, China

**Keywords:** exercise intervention, exercise components, autism spectrum disorder, repetitive stereotyped behaviors, meta-analysis

## Abstract

**Objective:**

To systematically evaluate the effect of exercise intervention and its components on repetitive stereotyped behaviors in patients with autism spectrum disorder (ASD).

**Methods:**

A computer-based search was conducted in PubMed, Web of Science, The Cochrane Library, and EMbase databases for randomized controlled trials (RCTs) related to exercise interventions for repetitive stereotyped behaviors in patients with ASD. The search covered all available data from the inception of each database until January 2025. Meta-analysis was performed using RevMan 5.4 software, methodological quality was assessed using the ROB scale, and publication bias was evaluated using Stata 17.0 software.

**Results:**

A total of 20 RCTs were included, comprising 671 patients with ASD. The meta-analysis results showed that exercise intervention had a positive effect on repetitive stereotyped behaviors in patients with ASD (SMD = −0.37, 95% CI: −0.52, −0.21, *p* < 0.05). Subgroup analysis results indicated that ball sports (SMD = −0.72, 95% CI: −1.09, −0.36, *p* < 0.001), longer duration (SMD = −0.55, 95% CI: −0.98, −0.12, *p* < 0.05), moderate to high frequency (SMD = −0.74, 95% CI: −1.05, −0.44, *p* < 0.001), longer time (SMD = −0.84, 95% CI: −1.26, −0.42, *p* < 0.001), and group participation (SMD = −0.48, 95% CI: −0.74, −0.21, p < 0.001) might show optimal dose–response relationships.

**Conclusion:**

Exercise intervention can effectively improve repetitive stereotyped behaviors in patients with ASD. The components of exercise intervention show a dose–response effect, with the best results likely occurring from ball sports, medium to long durations, moderate to high frequency, longer time, and group participation.

**Systematic review registration:**

The protocol for this meta-analysis has been registered in INPLASY, with the registration number INPLASY202520074.

## 1 Introduction

Autism Spectrum Disorder (ASD) is a complex neurodevelopmental disorder, characterized by core features including social communication deficits and restricted and repetitive behaviors (RRBs) (Lord et al., 2018). Repetitive behaviors encompass repetitive movements (e.g., hand-flapping, spinning), intensely focused interests, and rigid adherence to specific rules, all of which may lead to a decline in an individual’s ability to adapt to the environment and hinder the development of flexible behaviors (Leekam et al., 2011). In recent years, the global prevalence of ASD has been steadily increasing, with rates in developed countries reaching 1.5%, data from the Centers for Disease Control and Prevention (CDC) in 2021 indicate that the prevalence of ASD among children aged 0–8 has risen to 2.47% (Kim, 2013; Xu et al., 2018). However, there is currently no effective pharmacological intervention for the core symptoms of ASD. Existing treatments primarily focus on symptom management rather than fundamental improvement.

Exercise intervention not only promotes sensory integration and motor coordination but may also reduce the occurrence of stereotyped behaviors through behavioral substitution mechanisms (Wei et al., 2021). The structured characteristics of physical exercise, such as repetitiveness, predictability, and rhythmicity, resemble certain forms of stereotyped behaviors, allowing individuals with ASD to experience satisfaction from goal-directed activities, thereby reducing the frequency of non-goal-directed stereotyped behaviors (Moradi et al., 2020). For example, highly structured exercises such as karate training, swimming, and running have been shown to reduce repetitive movements (Movahedi et al., 2013). Additionally, Sensory-Motor Training, which enhances sensory integration and improves motor planning abilities, may also indirectly reduce the intensity of stereotyped behaviors (Xu et al., 2019). Simultaneously, including basic social etiquette in physical exercises, such as greeting peers, parents, or teachers during basketball training, or queuing, increases communication, physical contact, eye contact, and language among children with ASD, promoting social behaviors in these children (Wang et al., 2020). This, in turn, induces and strengthens social communication, improves gaze processing and attention, and ultimately enhances cognitive neural function, having a positive effect on brain function activation (Abujabra Merege Filho et al., 2014).

Currently, there are no objective, effective, and specific early diagnostic biomarkers or therapeutic drugs for the social impairments of ASD (Liu et al., 2016), and the medical needs remain unmet (Benedetti-Isaac et al., 2023). There are various treatment options available; however, the effectiveness of different rehabilitation methods varies, with issues such as high economic costs and low practicality. For example, speech therapy, occupational therapy, physical therapy, and behavioral interventions involve extremely high costs for families of children with ASD, placing a significant financial burden on individual families and social service agencies (Bremer et al., 2016). Exercise interventions have fewer side effects, lower economic costs, and are easier to implement, making them highly significant for promoting social adaptation in children. Although deficits in movement and physical activity are not core features of this disorder, addressing these deficits and increasing physical activity levels may provide more opportunities for social interaction with peers, improve attention and motor performance, and indirectly impact the core symptoms of ASD individuals (Srinivasan et al., 2014).

Although the effects of exercise interventions have been reported in several studies, the stability of these effects and the specific mechanisms remain controversial (Wei et al., 2021; Jia et al., 2023). A meta-analysis found that organized physical activities have a certain degree of improvement on stereotyped behaviors in individuals with ASD, but there are significant differences in intervention effects across different exercise modes (Sowa and Meulenbroek, 2012). Additionally, research by Wang et al. indicated that the impact of exercise on stereotyped behaviors may be individual-dependent, with significant variations in intervention effects, especially when different combinations of exercise intensity, duration, and frequency are used (Wang et al., 2022). However, current research has paid little attention to the dose–response relationship between exercise dosage and stereotyped behaviors, which limits the precise application of exercise interventions.

Previous meta-analyses on the effects of exercise interventions for repetitive stereotyped behaviors in individuals with ASD are limited, with most studies not addressing the “dose–response relationship” between exercise variables and repetitive behaviors. Therefore, this study aims to address the following research questions: “Can exercise improve repetitive stereotyped behaviors?” and “What exercise dosage is most effective in improving repetitive stereotyped behaviors?” This study plans to use meta-analysis methods to systematically review previous randomized controlled trials (RCTs), with stereotyped behaviors as the primary outcome measure. The study will explore the effects of various exercise variables, such as exercise mode (e.g., ball sports, equestrian training, sensory-motor training), exercise duration, frequency, time, and organizational format, on stereotyped behaviors, and attempt to develop an optimal exercise intervention program. Through systematic quantitative analysis, the study aims to provide more precise evidence-based guidance for future non-pharmacological interventions targeting stereotyped behaviors in ASD.

## 2 Research methods

### 2.1 Literature sources

The included literature was organized and analyzed according to the requirements of international guidelines for systematic reviews. The study adhered to the PRISMA (Preferred Reporting Items for Systematic Reviews and Meta-Analysis) statement and the Cochrane Handbook for Systematic Reviews of Interventions (Moher et al., 2009). The protocol for this meta-analysis has been registered in INPLASY, with the registration number INPLASY202520074.

Publicly available literature from international databases was retrieved, with the search period extending from the inception of the database to January 2025. A combination of controlled vocabulary and free text terms was used, including English search terms such as “Movement/Physical exercise/Physical activity/exercise/sport/Training/Physical Exercises/training/motion/activity/physical therapy/sport,” “autistic disorder/Autism Spectrum Disorders/Autistic Spectrum Disorders/Disorder, Autistic/Spectrum/Early Infantile Autism/Disorders, Asperger/Syndrome, Asperger,” “Stereotyped Behavior/Behaviors, Stereotyped/Behavior, Stereotyped/Stereotyped Behaviors,” and “randomized controlled trial/randomized/controlled/trial/randomized controlled trial/random/random allocation/RCT/RCTs,” to search the PubMed, Web of Science, EBSCOhost, and Cochrane Library databases. References from included studies and relevant reviews were also traced to ensure the comprehensiveness of the literature search. To ensure a comprehensive review of the literature, we also examined currently published relevant review articles and cross-referenced our included studies with the review lists to avoid omitting any pertinent research. The search strategy consisted of three key components: disease terms (Autism Spectrum Disorder, autism, Asperger’s syndrome, and autism spectrum), intervention terms (exercise therapy, exercise, physical activity, etc.), and study type (RCT).

### 2.2 Study selection based on the PICOS criteria

#### 2.2.1 Participants

This study included individuals of various ages, genders, and ethnicities. The enrolled ASD participants were diagnosed using the Diagnostic and Statistical Manual of Mental Disorders, 5th Edition (DSM-5), or other standardized diagnostic tools, such as the Autism Diagnostic Interview-Revised (ADI-R) and the Childhood Autism Rating Scale (CARS). Participants with other types of neurodevelopmental disorders, such as Attention Deficit Hyperactivity Disorder (ADHD) or psychiatric conditions, were excluded.

#### 2.2.2 Intervention

Given the unique nature of ASD treatment, participants were required to undergo regular rehabilitation therapy. Therefore, both the intervention and control groups in this study received regular rehabilitation training. The intervention group, in addition, received extra physical exercise interventions, regardless of the type of exercise. In contrast, the control group was strictly prohibited from receiving any exercise interventions beyond the prescribed rehabilitation therapy. This restriction was implemented to ensure that participants maintained their original lifestyle or received standard rehabilitation training without additional exercise interventions.

#### 2.2.3 Outcome measures

It is essential to use established and validated measurement tools to assess study outcomes. The primary outcome measures focused on evaluating the overall severity of autism in individuals, specifically assessing stereotyped behaviors using tools such as the Autism Treatment Evaluation Checklist (ATEC), the Childhood Autism Rating Scale (CARS), and the Autism Behavior Checklist (ABC), among others.

#### 2.2.4 Study types

We included randomized controlled trials (RCTs) published in peer-reviewed journals that assessed the effects of different types of physical exercise on individuals with ASD. We excluded non-randomized controlled trials, case reports or case series, systematic reviews and meta-analyses, reviews or letters, conference abstracts, dissertations, technical reports, scientific research projects, cell culture or animal studies, as well as studies published only as abstracts and certain cross-sectional study designs.

### 2.3 Inclusion and exclusion criteria

Inclusion Criteria: (1) Study type: Randomized controlled trials (RCTs); (2) Participants: Individuals diagnosed with ASD, or those who meet the diagnostic criteria for ASD according to the Fourth or Fifth Edition of the Diagnostic and Statistical Manual of Mental Disorders (DSM-IV or DSM-V); (3) Intervention: The control group receives standard rehabilitation therapy or no intervention, while the experimental group receives additional physical exercise on top of the standard rehabilitation; (4) Outcome measures: the primary or partial outcome measures must involve repetitive stereotyped behaviors.

Exclusion Criteria: (1) Reviews, commentaries, animal studies, duplicate publications, etc.; (2) Studies with unclear data descriptions, incomplete data, inability to retrieve raw data even after contacting the authors, data that cannot be transformed, or poor quality assessments; (3) Participants with other physical diseases; (4) Studies with unclear diagnostic criteria or intervention protocols.

### 2.4 Literature screening and data extraction

All studies retrieved from the online databases were imported into Endnote X9 software. Based on the predefined inclusion and exclusion criteria, two researchers (JY and RL) independently identified relevant studies by reading titles and abstracts. Subsequently, both authors conducted a full review of these studies to further filter them and cross-verify the final inclusion results. The two evaluators (JY and RL) extracted the characteristics of each study (title, authors, publication date, sample age, gender, country, sample size, experimental and control group details, assessment tools, measurement time points, outcome measures, intervention type and composition, duration, frequency, total number of treatment sessions) and entered them into a pre-designed Microsoft Excel sheet. We cross-checked the information to ensure consistency and accuracy in the data extraction process. Any discrepancies during the screening or data extraction process were resolved through discussion, and if necessary, a third researcher made the final decision through arbitration.

### 2.5 Quality assessment

In this part of the analysis, the risk of bias (ROB) for each included study was independently assessed by two authors using the Cochrane Collaboration tool (Higgins and Thompson, 2002), covering seven domains: random sequence generation, allocation concealment, participant and personnel blinding, outcome assessment blinding, incomplete outcome data, selective reporting, and other sources of bias. Each domain’s ROB was rated as low, unclear, or high risk of bias. Any discrepancies in quality assessment were resolved through consultation with a third author.

### 2.6 Evidence quality assessment

The GRADEprofiler system was used to assess the quality of evidence for outcome measures. The evaluation of evidence quality included five downgrading factors: publication bias, inconsistency, imprecision, indirectness, and risk of bias. The evidence was classified into four levels: high (no downgrade), moderate (one level downgrade), low (two levels downgrade), and very low (three levels downgrade) (Guyatt et al., 2008). The quality assessment was independently performed by two researchers. If discrepancies were found, a third researcher participated in the discussion until consensus was reached.

### 2.7 Statistical methods

All data analyses were performed using RevMan 5.4 Cochrane Collaboration (2020). For continuous variables, mean difference (MD) or standardized mean difference (SMD) was used for analysis. When different measurement tools or units were used for the same outcome, the SMD with a 95% confidence interval (CI) was selected as the combined statistic. In contrast, when the same assessment tool was used across studies, the MD with a 95% CI was calculated. If we encountered continuous data with opposite directions of effect (i.e., higher scores represent better outcomes in some studies, while lower scores represent better outcomes in others), we applied an inverse approach to data processing by reversing the mean and standard deviation before combining the results again. To assess the heterogeneity among study results, we used the *I*^2^ statistic. The *I*^2^ statistic values of 75, 50, and 25% were considered thresholds for high, moderate, and low heterogeneity, respectively (Higgins and Thompson, 2002). When *I*^2^ > 50%, a random effects model was applied, and sensitivity analysis was used to explore the sources of heterogeneity; when *I*^2^ ≤ 50%, heterogeneity among the studies was considered acceptable (Heissel et al., 2023). The “Stata 18.0” software was used to detect publication bias, with funnel plots being used to assess the possibility of publication bias (Egger et al., 1997). Egger’s test was used to detect publication bias, with the 95% confidence interval serving as the outcome measure for the meta-analysis (Begg and Mazumdar, 1994). If the test results indicated publication bias, the “trim-and-fill” method was used to assess the stability of the meta-analysis results (Duval and Tweedie, 2000). If fewer than 10 studies were included, publication bias was not assessed to avoid reducing the power of the test (Chen and Zhu, 2024). The significance level for the meta-analysis was set at *p* < 0.05 (Jakobsen et al., 2014). For studies that reported sample size, mean, and standard error, the standard error was converted to standard deviation after data extraction. When the data were in the opposite direction (i.e., larger values indicate worse outcomes, or smaller values indicate better outcomes), the mean was multiplied by −1 according to the recommendations of the Cochrane Handbook (Nasser, 2020; Owen et al., 2020). We further conducted subgroup analyses based on the characteristics of the interventions, including the type of exercise, duration, frequency, and mode of participation. Additionally, subgroup analyses were performed based on the age of the participants. Sensitivity analyses were conducted to assess the robustness of our findings, including excluding individual studies to evaluate their impact on the overall results. If more than 10 studies were included in the meta-analysis, funnel plots were used to assess publication bias. We contacted the study authors via email to obtain missing data. When missing data were unavailable, we only analyzed the reported data.

## 3 Results

### 3.1 Search results

**Figure 1:**
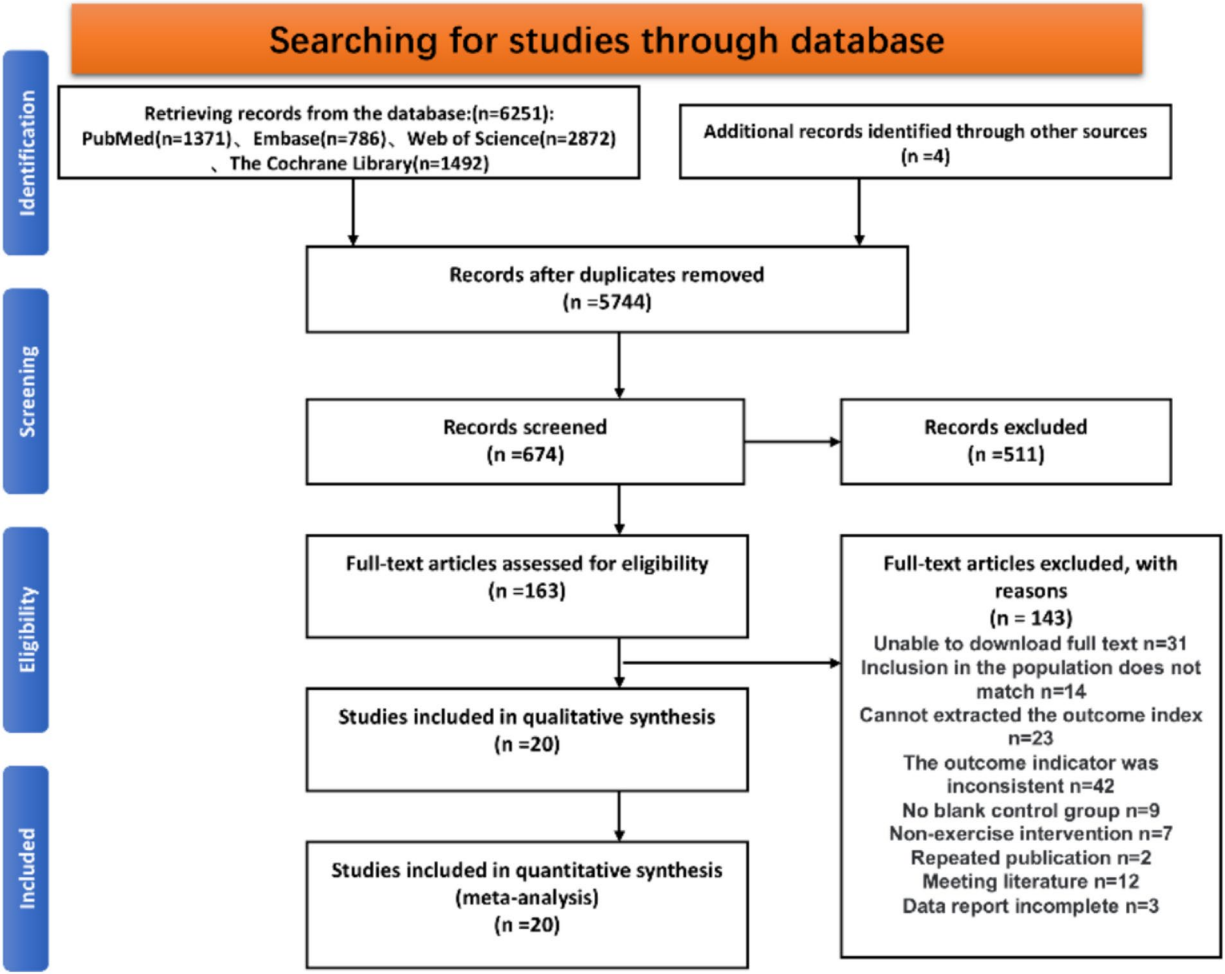
Selection of study and screening flowchart as required by the PRISMA statement.

A total of 6,255 articles were retrieved through PubMed, EMbase, Web of Science, and The Cochrane Library, supplemented by 4 articles from prior literature. The articles were imported into Endnote X9, and after removing duplicates, 5,744 articles remained. A preliminary screening based on titles and abstracts yielded 163 articles, and after full-text review, 143 articles were excluded. Exclusions were made for the following reasons: 31 articles could not be accessed in full, 14 articles did not meet the inclusion criteria for participants, 23 articles lacked outcome measures, 42 articles had incompatible outcome measures, 16 articles did not match the intervention type, 2 articles were duplicates, 12 were conference papers, and 3 had incomplete data reporting. A total of 20 studies were included in the final analysis. The detailed literature selection flowchart is shown in Figure 1.

### 3.2 Basic information of included studies and intervention characteristics

A total of 20 studies (671 participants) were included (Table 1), all of which involved participants diagnosed with Autism Spectrum Disorder (ASD). The studies were published between 2013 and 2024. For all included studies, the sample size was based on the number of participants at the time of outcome measure assessment, and there were no significant differences in baseline values. Among these, 6 studies were from China, 5 from the United States, 2 from Iran, 2 from the United Kingdom, 2 from Switzerland, 2 from Italy, and 1 from Turkey. Table 1 shows that all included studies reported either full or partial details of the exercise variables, including the intervention type, exercise duration, frequency, participation format, and the control group intervention methods. The exercise types included five different categories: 4 studies (20%) used ball sports, 5 studies (25%) used equestrian activities, 4 studies (20%) used multi-component exercise combining various activities, 4 studies (20%) involved martial arts/dance, and 3 studies (15%) used water sports.

**Table 1:** Basic features of literature included in the study.

Literature included	Countries	Sample E(male/female)/C(male/female)	Age(years)		Intervention type		Intervention dose				Outcome index
			Experimental group	Control group	Experimental group	Control group	Period Week	Frequency Times/week	Time Min/time	Organizational form	
Chan et al. (2013)	China	20(NR)/20(NR)	11.28 ± 3.90	12.42 ± 3.25	NYG + Muscle relaxation	Muscle relaxation	4	2	60	I	ATEC
Haghighi et al. (2022)	Iran	8(5–3)/8(4–4)	9.00 ± 1.31	8.13 ± 1.36	CPT	No intervention	8	NR	60 ~ 70	II	GARS-2
Harris and Williams (2017)	Britain	10(9–1)/14(12–2)	7.96 ± 0.78	6.97 ± 0.33	THR	No intervention	7	NR	45	I	CARS-2
Tse (2020)	China	15(13–2)/12(10–2)	10.07 ± 1.10	9.42 ± 0.90	CPT	Daily activities	12	4	30	II	CBCL
Coman et al. (2018)	America	25(23–2)/25(19–6)	8.70 ± 1.60	8.70 ± 1.60	THR	Daily activities	12	1	70	I	SRS
Moradi et al. (2020)	Iran	25/25	1.03 ± 7.64	1.25 ± 7.20	SIT	Daily activities	8	2	50	I	GARS-2
Marzouki et al. (2022)	Switzerland	8(7–1)/6(5–1)	6.3 ± 0.50	6.3 ± 0.50	AT	Daily activities	8	2	50	II	GARS-2
Marzouki et al. (2022)	Switzerland	8(8–0)/6(5–1)	6.4 ± 0.50	6.3 ± 0.50	AT	Daily activities	8	2	50	III	GARS-2
Phung and Goldberg (2021)	America	14(14–0)/20(144–6)	9.10 ± 1.10	9.52 ± 1.07	MMAT	Daily activities	13	2	45	I	SSIS
Wang et al. (2020)	China	18(15–3)/15(13–2)	5.11 ± 0.65	4.70 ± 0.70	MBTP	Daily activities	12	5	40	II	RBS-R
Cai K. et al. (2020)	China	15(NR)/14(NR)	5.13 ± 0.61	4.68 ± 0.72	MBTP	Daily activities	12	5	40	II	SRS-2
Cai K.-L. et al. (2020)	China	30(28–2)/29(27–3)	4.56 ± 0.84	5.03 ± 0.64	MBTP	Daily activities	12	5	40	II	SRS-2
Bass et al. (2009)	America	19(17–2)/15(13–2)	6.95 ± 1.67	7.73 ± 1.65	THR + Conventional treatment	Conventional treatment	12	1	60	II	SRS
Zanobini and Solari (2019)	Italy	13(10–3)/12(9–3)	5.69 ± 1.27	5.42 ± 1.54	AT + Conventional treatment	Conventional treatment	24	0.5	30	III	ABC
Gabriels et al. (2015)	America	58(49–9)/58(52–6)	10.5 ± 3.20	10.0 ± 2.70	THR	Daily activities	10	NR	45	I	VABS-II
Yang et al. (2021)	China	15(21–3)/15(13–2)	5.03 ± 0.55	4.67 ± 0.70	MBTP + Routine rehabilitation	Routine rehabilitation	12	5	40	II	SRS-2
Aithal et al. (2021)	Britain	10(NR)/16(NR)	11.53	9.77	DMP + Routine nursing	Routine nursing	5	2	40	II	SCQ
Bahrami et al. (2012)	Italy	15(NR)/15(NR)	9.20 ± 3.32	9.06 ± 3.33	Kata techniques	Daily activities	14	7	90	I	GARS-2.
Pan et al. (2018)	America	8(6–2)/8(7–1)	11.88 ± 2.45	9.80 ± 2.82	THR	Daily activities	10	NR	45	I	ABC-C
Gabriels et al. (2015)	Turkey	17(14–3)/17(6–1)	4.838 ± 0.733	4.688 ± 0.748	CPT	No intervention	12	2	60	III	GARS-2

For convenience, only the first author is given. NR means not reported, and E(male/female)/C(male/female) means experimental group (male/female)/control group (male/female). Individual exercise with group participation refers to individual exercise where group activities accounted for more than 50% of the total duration. (I: individual exercise; II: group exercise; III: individual exercise with group participation). THR, therapeutic riding; MBTP, Mini Basketball Training; DMT, DMP, dance movement; CPT, comprehensive physical training; AT, spa training; SIT, sensory integration training; NYG, Chinese traditional mind–body movement inner Yang Gong in the Zen form; MMAT, Mixed martial Arts Training; KD, karate training; Social Response Scale 2nd Edition (SRS-2), Repetitive Behavior Scale Revision (RBS-R), Social Interaction Questionnaire (SCQ), Adaptive Behavior Scale 2nd Edition (VABS-II), Social Skills Improvement System (SSIS), Children’s Emotional and Behavioral Functioning (CBCL), Autism Behavior Checklist (ABC), Autism Rating Scale (GARS-2), Childhood Autism Rating scale (CARS), Autism Treatment Evaluation Checklist (ATEC).

### 3.3 Quality assessment of included studies

**Figure 2:**
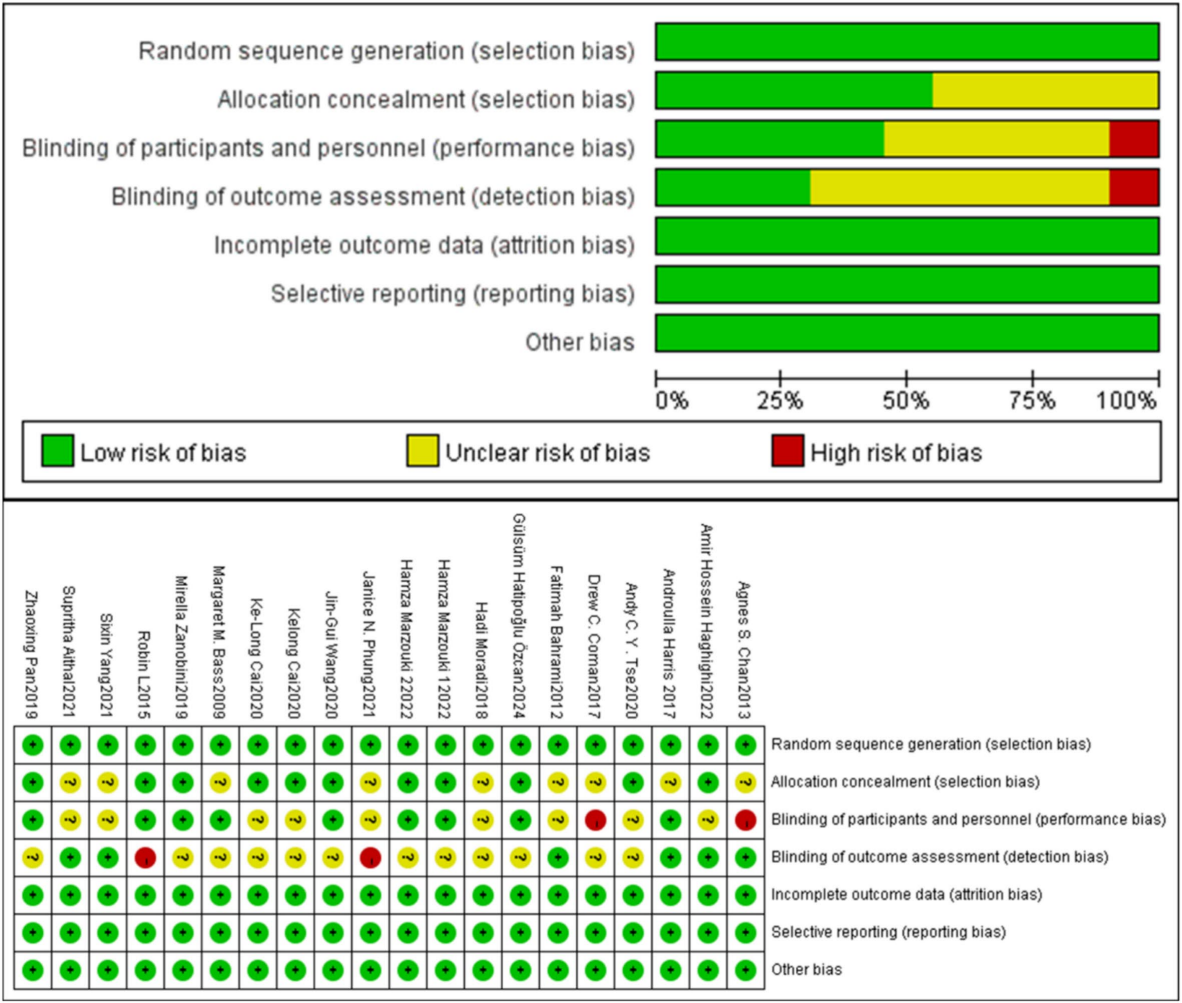
Quality assessment of the included studies.

In our analysis, some studies were rated as having low risk in terms of random sequence generation (Selection Bias) and allocation concealment (Allocation Concealment). Specifically, studies by Chan et al. (2013), Haghighi et al. (2022), Harris and Williams (2017), Tse (2020), Özcan et al. (2024), Cai K.-L. et al. (2020), and others were considered to have lower risks of selection bias and allocation concealment. These studies reported using randomization procedures (such as computer software or random number tables) and transparent allocation methods (such as sealed envelopes). However, some studies [such as Coman et al. (2018), Bahrami et al. (2012), Gabriels et al. (2015), etc.] were considered at higher risk due to insufficient explanation of allocation concealment, which could not reduce the related bias risks. Additionally, regarding participant and outcome assessment blinding (Performance Bias and Detection Bias), although many studies were unable to implement effective participant blinding due to the nature of the exercise interventions, some studies reported blinding procedures. For example, Phung and Goldberg (2021), Zanobini and Solari (2019), and others implemented blinding during outcome assessment, while other studies did not provide detailed information on blinding, leading to unclear risk of bias in the assessments. Regarding outcome data integrity (Incomplete Outcome Data), most studies employed rigorous data analysis methods and did not report missing data or participant dropout, showing low risk. For example, studies by Bass et al. (2009), Yang et al. (2021), and others clearly used intention-to-treat (ITT) analysis and did not report missing data, ensuring the integrity of the results. However, some studies did not clearly report how missing data or participant dropouts were handled. Overall, none of the included studies reported other potential bias issues, indicating good study design and implementation. Detailed risk bias assessment results for each study are shown in Figure 2. These findings reflect the importance of randomization and blinding procedures in reducing potential biases when assessing study quality. Future studies should aim to improve transparency and detail in these areas.

### 3.4 Meta-analysis results

#### 3.4.1 Overall effectiveness score for stereotyped behavior

A total of 20 studies (671 participants) were included to compare the effects of physical exercise on stereotyped behaviors in individuals with Autism Spectrum Disorder (ASD). Figure 1 shows the meta-analysis results for social interaction disorders in the exercise group. The heterogeneity test results showed *X*^2^ = 28.62, *I*^2^ = 34%, *p* = 0.07, indicating low statistical heterogeneity between studies, so a fixed-effect model was used for the analysis. The meta-analysis results showed a combined effect size of SMD = −0.37, 95% CI: −0.52, −0.21, *p* < 0.05, indicating a statistically significant difference. This suggests that physical exercise can effectively improve stereotyped behaviors in individuals with Autism Spectrum Disorder (ASD) compared to the control group.

#### 3.4.2 Subgroup analysis

A subgroup analysis based on intervention type was conducted. The combined effect sizes showed: ball games (SMD = −0.72, 95% CI: −0.52, −0.21, *p* < 0.001); equestrian exercise (SMD = −0.18, 95% CI: −0.44, 0.08, *p* > 0.05); multi-component exercise (SMD = −0.41, 95% CI: −0.76, −0.05, *p* < 0.05); martial arts/dance (SMD = −0.46, 95% CI: −0.83, −0.10, *p* < 0.05); aquatic exercise (SMD = −0.18, 95% CI: −0.71, 0.34, *p* > 0.05). Heterogeneity tests showed: for ball games, *I*^2^ = 0%, *p* = 0.73; for equestrian exercise, *I*^2^ = 68%, *p* = 0.01; for multi-component exercise, *I*^2^ = 22%, *p* = 0.28; for martial arts/dance, *I*^2^ = 20%, *p* = 0.29; for aquatic exercise, *I*^2^ = 0%, *p* = 0.80. The results showed that ball games, multi-component exercise, and martial arts/dance had a significant improvement compared to the control group, while equestrian exercise and aquatic exercise showed no significant difference compared to the control group. Equestrian exercise exhibited moderate heterogeneity, while the remaining exercise types had low heterogeneity (Table 2).

**Table 2:** Summary of meta-analysis results.

Moderator variables		*X*^2^ (df)	*I*^2^%	*n*(ES)	ES, 95%CI	*P*值
Combined effect size		28.62(19)	34	20	SMD = −0.37[−0.52, −0.21]	<0.001
Type of exercise	Ball sports	1.29(3)	0	4	SMD = −0.72[−1.09, −0.36]	<0.001
	Equestrian	12.69(4)	68	5	SMD = −0.18[−0.44, 0.08]	0.17
	Multicomponent exercise	3.84(3)	22	4	SMD = −0.41[−0.76, −0.05]	0.03
	Martial arts/dance	3.77(3)	20	4	SMD = −0.46[−0.83, −0.10]	0.01
	Water sports	0.44(2)	0	3	SMD = −0.18[−0.71, 0.34]	0.049
Duration (weeks)	≤8 Weeks	2.31(6)	0	7	SMD = −0.18[−0.48, 0.11]	0.22
	8–12 Weeks	20.43(9)	56	10	SMD = −0.42[−0.62, −0.22]	<0.001
	>12 Weeks	3.48(2)	42	3	SMD = −0.55[−0.98, −0.12]	0.01
Frequency (Sessions/week)	Low frequency ≤3	16.99(9)	47	10	SMD = -0.27[−0.49, −0.04]	0.02
	Medium-high f>3	1.69(5)	0	6	SMD = -0.74[−1.05, −0.44]	<0.001
	Not reported	1.93(3)	0	4	SMD = -0.19[−0.49, 0.12]	0.23
Exercise duration (minutes)	Short duration (≤45 min)	13.01(10)	23	12	SMD = -0.42[−0.63, −0.22]	<0.001
	Medium duration (45–60 min)	4.91(5)	0	6	SMD = −0.03[−0.32, 0.26]	0.84
	Long duration (>60 min)	0.47(2)	0	3	SMD = −0.84[−1.26, −0.42]	<0.001
Participation format	Individual activity	11.26(7)	38	8	SMD = −0.34[−0.55, −0.13]	0.002
	Group activity	15.53(8)	48	9	SMD = -0.48[−0.74, −0.21]	<0.001
	Individual participation in group	0.56(2)	0	3	SMD = −0.19[−0.66, 0.27]	0.41

Subgroup analysis was conducted based on the exercise intervention duration. The combined effect sizes showed: short duration (SMD = −0.18, 95% CI: −0.48, 0.11, *p* > 0.05); medium duration (SMD = −0.42, 95% CI: −0.62, −0.22, *p* < 0.001); long duration (SMD = −0.55, 95% CI: −0.98, −0.12, *p* < 0.05). The heterogeneity test results showed: short duration (*I*^2^ = 0%, *p* = 0.22); medium duration (*I*^2^ = 56%, *p* = 0.02); long duration (*I*^2^ = 42%, *p* = 0.18). The results indicate that medium and long duration interventions significantly improved outcomes compared to the control group, while the short duration intervention showed no significant improvement. The medium duration showed moderate heterogeneity, while both short and long durations exhibited low heterogeneity (Table 2).

Subgroup analysis was conducted based on the exercise intervention frequency. The combined effect sizes showed: low frequency (SMD = −0.42, 95% CI: −0.49, −0.04, *p* < 0.05); moderate-high frequency (SMD = −0.74, 95% CI: −1.05, −0.44, *p* < 0.001); unreported frequency (SMD = −0.19, 95% CI: −0.49, 0.12, *p* > 0.05). The heterogeneity test results showed: low frequency (*I*^2^ = 47%, *p* = 0.05); moderate-high frequency (*I*^2^ = 0%, *p* = 0.89); unreported frequency (*I*^2^ = 0%, *p* = 0.59). The results indicate that moderate-high frequency exercise significantly improved outcomes compared to the control group, while low frequency exercise showed no significant improvement. Low frequency showed moderate heterogeneity, whereas moderate-high frequency and unreported frequency exhibited low heterogeneity (Table 2).

Subgroup analysis was conducted based on the exercise intervention duration. The combined effect sizes showed: short duration (SMD = −0.42, 95% CI: −0.63, −0.22, *p* < 0.001); medium duration (SMD = −0.03, 95% CI: −0.32, 0.26, *p* > 0.05); long duration (SMD = −0.84, 95% CI: −1.26, −0.42, *p* < 0.001). The heterogeneity test results showed: short duration (*I*^2^ = 23%, *p* = 0.22); medium duration (I^2^ = 0%, *p* = 0.43); long duration (*I*^2^ = 0%, *p* = 0.79). The results indicate that both short and long duration interventions significantly improved outcomes compared to the control group, while medium duration showed no significant improvement. Heterogeneity across different intervention durations was low (Table 2).

Subgroup analysis was conducted based on the exercise organization format. The combined effect sizes showed: individual exercise (SMD = −0.34, 95% CI: −0.55, −0.13, *p* < 0.05); group exercise (SMD = −0.48, 95% CI: −0.74, −0.21, *p* < 0.001); individual events with multiple participants (SMD = −0.19, 95% CI: −0.66, 0.27, *p* > 0.05). The heterogeneity test results showed: individual exercise (*I*^2^ = 38%, *p* = 0.13); group exercise (*I*^2^ = 48%, *p* = 0.05); individual events with multiple participants (*I*^2^ = 0%, *p* = 0.75). The results indicate that both individual exercise and group exercise significantly improved outcomes compared to the control group, whereas individual events with multiple participants did not show significant improvement compared to the control group. The heterogeneity across different organization formats was low (Table 2).

Subgroup analysis was conducted based on age groups. The combined effect sizes showed: preschool age (SMD = −0.30, 95% CI: −0.53, −0.06, *p* < 0.05); school-age (SMD = −0.42, 95% CI: −0.63, −0.22, *p* < 0.001). The heterogeneity test results showed: preschool age (*I*^2^ = 39%, *p* = 0.10); school-age (*I*^2^ = 32%, *p* = 0.51). The results indicate that both preschool and school-age groups showed significant improvement compared to the control group. The heterogeneity for both preschool and school-age groups was low (Table 2).

### 3.5 Sensitivity analysis

**Figure 3:**
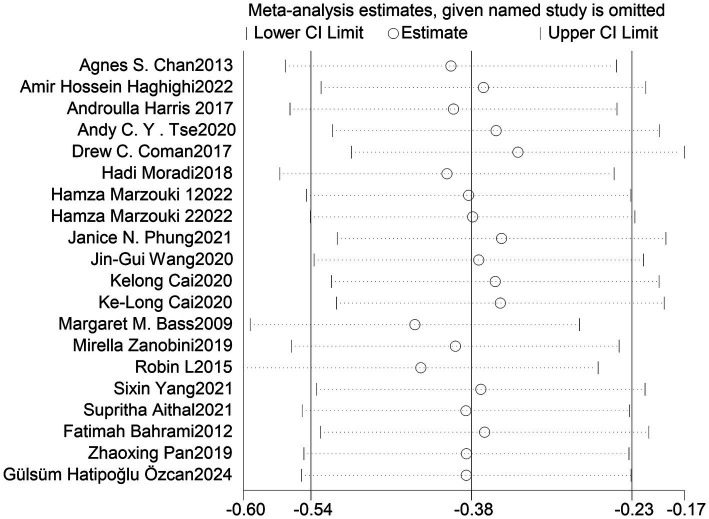
Confidence interval distribution after excluding individual studies.

To explore whether the heterogeneity between studies is caused by a single study, a sensitivity analysis was conducted by sequentially removing each study and reanalyzing the combined effect. After removing each study, the range of the combined effect size (SMD) was from −0.42 to −0.32, the range of *I*^2^ was from 10 to 37%, and *p*-values were all <0.05. Although the heterogeneity decreased by 29% after removing the Margaret M. B. 2009 study, it did not affect the outcomes, and removing the other studies one by one did not lead to any changes in the results. The results indicate that the sensitivity of the data in this study is low, and there was no substantial change in the results from the meta-analysis, suggesting that the findings have a certain level of stability and reliability (Table 3 and Figure 3).

**Table 3:** The pooled effect of social communication function after excluding a single study.

Scoring types and included studies	SMD	95%CI	*P* (the pooled effect)	*I*^2^%
Chan et al. (2013)	−0.39	−0.55, −0.23	*P* < 0.001	35
Haghighi et al. (2022)	−0.36	−0.52, −0.20	*P* < 0.001	35
Harris and Williams (2017)	−0.39	−0.54, −0.23	*P* < 0.001	34
Tse (2020)	−0.35	−0.50, −0.19	*P* < 0.001	32
Coman et al. (2018)	−0.32	−0.49, −0.16	*P* < 0.001	26
Moradi et al. (2020)	−0.39	−0.55, −0.23	*P* < 0.001	35
Marzouki et al. (2022)	−0.37	−0.53, −0.21	*P* < 0.001	37
Marzouki et al. (2022)	−0.37	−0.53, −0.21	*P* < 0.001	37
Phung and Goldberg (2021)	−0.34	−0.50, −0.18	*P* < 0.001	31
Wang et al. (2020)	−0.36	−0.52, −0.20	*P* < 0.001	37
Cai K. et al. (2020)	−0.35	−0.50, −0.19	*P* < 0.001	33
Cai K.-L. et al. (2020)	−0.34	−0.50, −0.18	*P* < 0.001	30
Bass et al. (2009)	−0.42	−0.58, −0.26	*P* < 0.001	10
Zanobini and Solari (2019)	−0.39	−0.54, −0.23	*P* < 0.001	35
Gabriels et al. (2015)	−0.42	−0.59, −0.24	*P* < 0.001	33
Yang et al. (2021)	−0.36	−0.52, −0.20	*P* < 0.001	37
Aithal et al. (2021)	−0.37	−0.53, −0.22	*P* < 0.001	37
Bahrami et al. (2012)	−0.36	−0.52, −0.20	*P* < 0.001	36
Pan et al. (2018)	−0.37	−0.53, −0.22	*P* < 0.001	37
Gabriels et al. (2015)	−0.37	−0.53, −0.21	*P* < 0.001	37

### 3.6 Publication bias

**Figure 4:**
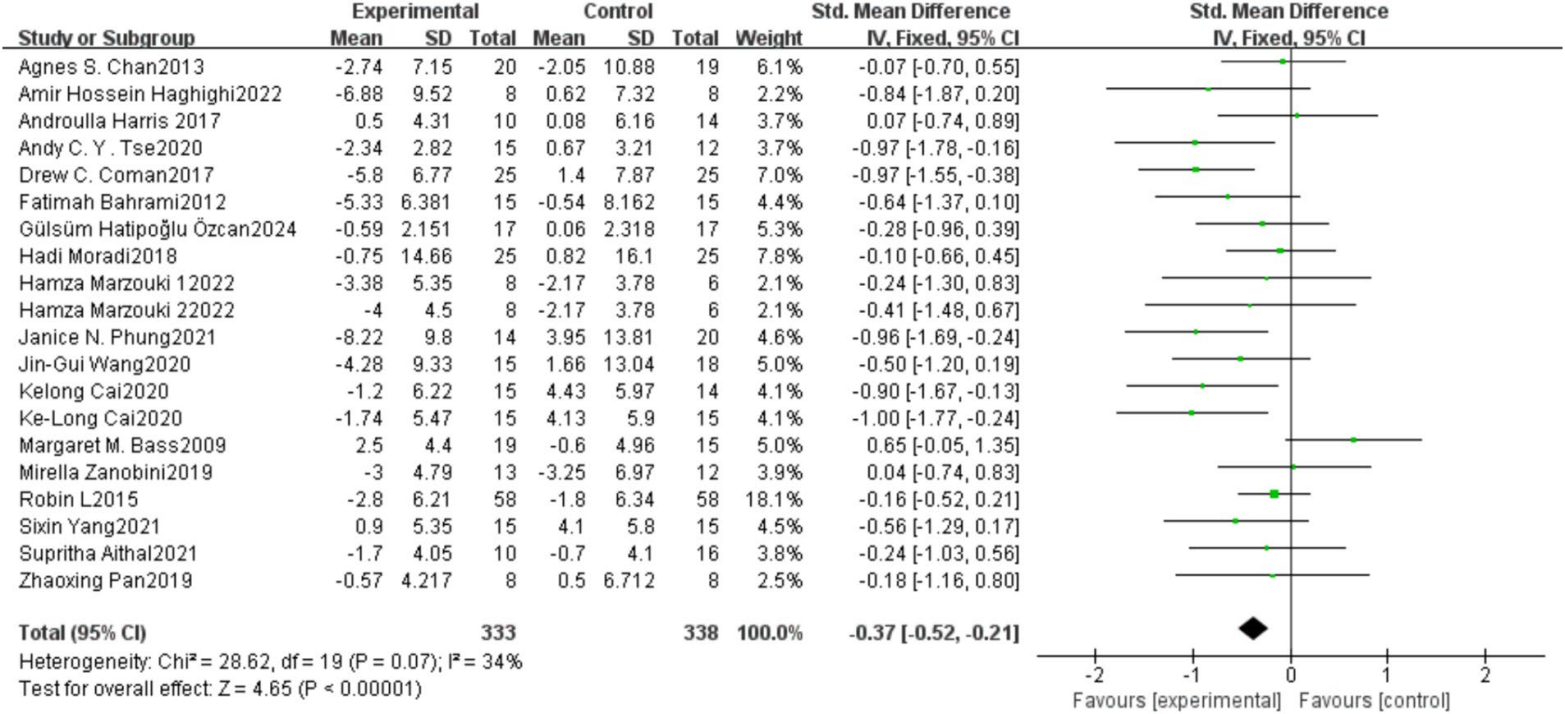
Forest plot of the meta-analysis on the effect of physical exercise on repetitive behaviors in individuals with autism spectrum disorder.

**Figure 5:**
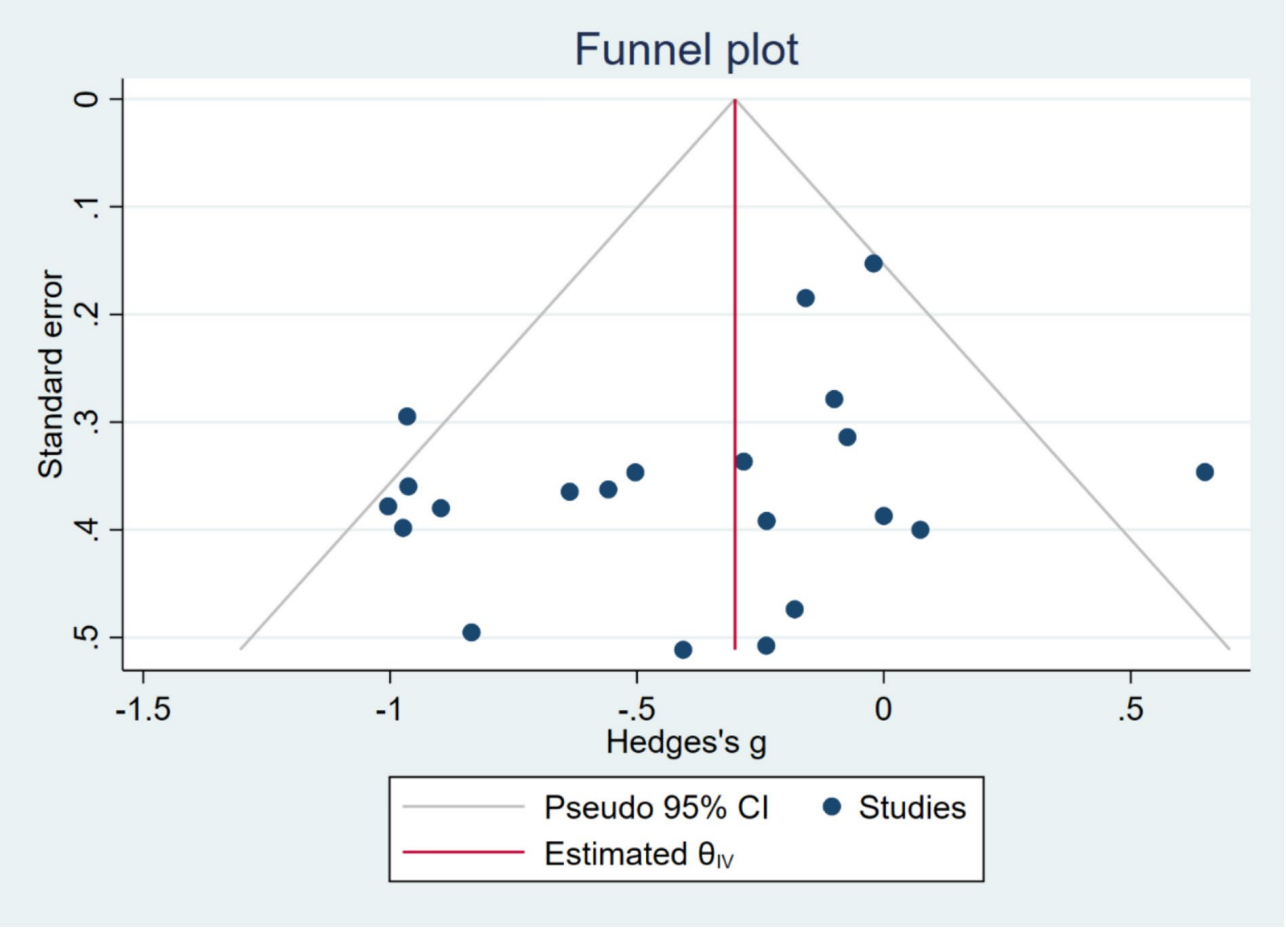
Egger’s test results.

Egger’s Test showed that there was no significant difference, with *z* = −1.33 (continuity corrected), Pr > |*z*| = 0.183 (continuity corrected) > 0.05 (Figure 4). Bias testing could not be performed for other individual studies, which may carry some bias. However, the overall test results suggest that this study does not exhibit publication bias, and the findings are relatively robust (Figure 5).

### 3.7 Quality of evidence assessment

Used GRADEprofiler for the assessment of evidence quality, no downgrading factors such as publication bias, inconsistency, imprecision, indirectness, or risk of bias were identified. The evidence quality evaluating the effect of physical exercise on improving repetitive stereotyped behaviors in children with ASD was rated as high.

### 3.8 Adverse events due to exercise

Across all studies included in this analysis, there were no reports of any adverse events.

## 4 Discussion

### 4.1 Summary of results

#### 4.1.1 The impact of exercise modalities

This study found that ball sports, multi-component physical exercise, and martial arts/dance had significant effects in reducing stereotypical behaviors in children with ASD, while equestrian and aquatic sports did not show significant intervention effects. Ball sports and martial arts/dance typically involve higher levels of movement complexity and social interaction. For example, basketball and soccer require individuals to continuously adjust their movement strategies in dynamic environments, promoting executive function and cognitive flexibility (Diamond, 2013). Moreover, martial arts and dance emphasize imitation learning and rhythmic perception, which may enhance sensory integration abilities, thereby reducing repetitive behaviors (Phung and Goldberg, 2021; Aithal et al., 2021). In contrast, equestrian and aquatic sports did not show significant effects on stereotypical behaviors, which may be related to the movement structures and sensory feedback characteristics of these activities. Although equestrian sports can improve individuals’ balance and emotional regulation (Harris and Williams, 2017), their primary benefits may be more focused on social communication and emotional stability rather than reducing stereotypical behaviors. Aquatic sports, on the other hand, may reduce proprioceptive input due to the buoyancy of water, which could lead to a failure to break the dependency on repetitive movement patterns effectively (Marzouki et al., 2022).

#### 4.1.2 The impact of intervention duration on the improvement of stereotypical behaviors

In the subgroup analysis based on intervention duration, we found that interventions lasting 8 weeks or less had no significant effects, whereas interventions lasting 8–12 weeks or more effectively reduced stereotypical behaviors, with a higher effect size as the intervention duration increased. This trend may reflect the cumulative effect of exercise in improving stereotypical behaviors, meaning that individuals require a longer period for adaptation and practice to form new movement patterns and reduce reliance on stereotypical behaviors (Botvinick and Braver, 2015). Additionally, longer interventions may be more conducive to changes in neuroplasticity, particularly in brain areas related to executive functions, such as the prefrontal cortex and basal ganglia (Wei et al., 2022).

#### 4.1.3 The role of intervention frequency and session duration

Regarding intervention frequency, we found that interventions of ≤3 sessions per week and >3 sessions per week were both effective, but the intervention effects improved with increased frequency. This finding is consistent with previous research, suggesting that higher intervention frequencies may enhance exercise-related neural adaptation, improve motor execution, and reduce children with ASD’s reliance on stereotypical behaviors (Bremer et al., 2016). Regarding session duration, we found that interventions of ≤45 min were effective, those of 45–60 min were ineffective, and interventions lasting >60 min showed significant effects again. This non-linear pattern may be related to the attention-maintenance capacity and fatigue state of children with ASD. Shorter-duration exercise (≤45 min) may be sufficient to provide sensory input and motor training, while 45–60-min sessions may lead to increased fatigue and reduced attention, thus weakening the intervention effect (Afshari, 2012). However, interventions lasting more than 60 min may involve more comprehensive exercise patterns, including diverse sensory inputs and longer durations of repetitive practice, ultimately enhancing the substitution effect on stereotypical behaviors (Jia et al., 2021).

#### 4.1.4 The impact of participation format: individual vs. group

Our study shows that both individual and group exercises can reduce stereotypical behaviors in children with ASD, but group exercise has a more significant effect. This may be because group exercise not only provides goal-directed movements but also increases social engagement opportunities, thereby reducing children’s reliance on stereotypical behaviors (Wang et al., 2020). For example, team sports require individuals to continuously adjust their behavior to meet the demands of teamwork, and this change may foster the development of flexible thinking, reducing reliance on repetitive stereotypical behaviors (Yang et al., 2021).

### 4.2 Comparison to similar studies

Compared to previous studies, our results show some differences in terms of exercise type, intervention duration, frequency, and time. To better understand these differences, we compared our findings with previous studies of a similar nature and discussed potential influencing factors. Through comparison with previous research, we found that our study results show differences in exercise type, intervention duration, frequency, time, and participation format. These differences may be related to variations in exercise modalities, sensory input, the degree of social interaction, and the specific design of the intervention programs.

#### 4.2.1 Why are ball sports and martial arts effective, but equestrian and water sports ineffective?

This study found that ball sports, martial arts/dance, and multi-component physical exercise had significant effects in reducing stereotypical behaviors, while equestrian and water sports did not show significant improvement. However, some studies hold a different view on this result. For example, Lang et al. (2010) in their systematic review pointed out that equestrian training had a positive impact on emotional regulation and social communication in individuals with ASD, but did not explicitly mention improvements in stereotypical behaviors. Pan (2010) found that swimming training effectively improved social behaviors and motor skills in children with ASD, but did not observe significant effects on stereotypical behaviors.

These differences may arise from the following factors: (1) complexity of exercise modalities. Ball sports and martial arts/dance typically involve multi-task processing, requiring individuals to continuously adjust their movements, focus on goals, and interact with the environment or others. For example, basketball requires continuous adjustments in positioning, passing, and receiving, significantly reducing the occurrence of repetitive stereotypical behaviors. Equestrian and water sports may be too stable and passive. Equestrian training mainly relies on the rhythm of the horse’s movements, while swimming reduces exercise load due to the buoyancy of water, which may allow individuals to maintain their original stereotypical behavior patterns. (2) Differences in sensory input. Ball sports and martial arts/dance involve strong proprioception and vestibular input, which help enhance sensory integration. Sensory integration deficits are considered a key factor contributing to stereotypical behaviors in individuals with ASD. However, the sensory input in equestrian sports is relatively singular, mainly providing vestibular stimulation, and cannot integrate multiple sensory channels comprehensively, which may make it difficult to reduce stereotypical behaviors. (3) Social interaction factors: Team ball sports and martial arts training typically require interaction with peers, while equestrian and swimming are more individual activities, offering fewer opportunities for social interaction. This may result in weaker effects in reducing stereotypical behaviors.

#### 4.2.2 Why are interventions below 8 weeks ineffective, while longer interventions are more effective?

This study found that interventions shorter than 8 weeks did not show significant improvement, while interventions lasting 8–12 weeks and over 12 weeks effectively reduced stereotypical behaviors. In contrast, the meta-analysis by Srinivasan et al. (2016) pointed out that some 6–8 week physical interventions had shown improvements in social skills and repetitive stereotypical behaviors in children with ASD. This difference may arise from the following factors: (1) Neuroplasticity and cumulative effects. The reshaping of the nervous system through exercise requires a longer period of time. Existing studies have shown that physical exercise can enhance the connectivity between the prefrontal cortex and basal ganglia, thereby improving behavioral regulation (Botvinick and Braver, 2015). This neuroplasticity effect typically manifests after 8 weeks, whereas short-term interventions may not be sufficient to produce significant effects. Previous studies on short-term interventions may have focused on social skills rather than stereotypical behaviors, and reducing stereotypical behaviors might require more prolonged sensory integration training and executive function enhancement. (2) Differences in exercise programs: Some previous studies on short-term interventions focused on low-intensity or single forms of exercise, such as mild yoga or simple walking training. These interventions may not have been sufficient to produce significant effects on stereotypical behaviors. In contrast, this study included more challenging sports, such as ball sports and martial arts training, which may be one reason for the differences in the intervention period outcomes.

#### 4.2.3 Why are high frequency and longer duration exercises more effective?

This study found that interventions of ≥3 sessions per week were more effective, with durations of ≤45 min and >60 min showing effectiveness, while interventions lasting 45–60 min were ineffective. However, the study by Bremer et al. (2016) argued that interventions lasting 45–60 min can effectively reduce stereotypical behaviors in individuals with ASD. Possible reasons for this discrepancy include: (1) The non-linear effect of exercise intensity. Within the 45–60 min time window, individuals with ASD may experience motor fatigue and attention decrement, which may lead to less noticeable improvements in stereotypical behaviors (Afshari, 2012). However, exercise durations exceeding 60 min may involve more social interaction and multi-task training, reactivating the prefrontal cortex and enhancing the substitution effect for stereotypical behaviors. (2) Psychological factors. The adaptability of children with ASD to exercise may vary due to individual differences. Some individuals may experience an adaptation phase at the beginning of the exercise. If the transition period is too short (e.g., 45–60 min), the intervention may not yield significant effects, while longer durations of exercise may help them reach a more efficient state (Jia et al., 2021).

#### 4.2.4 Why are team sports more effective than individual sports?

Previous research has shown conflicting results regarding the effects of individual vs. team sports on the improvement of stereotypical behaviors. Jia et al. (2023) found that individual sports resulted in some improvement for children with ASD, but the effects were not as significant as those of team sports. Other studies (Blair and Raver, 2015) found that while individual sports can provide sensory input, they lack social interaction, which is an important factor in reducing stereotypical behaviors. The results of this study support the advantages of team sports, which may be related to the following factors: (1) Increased social cognitive load. Team sports require individuals with ASD to continually process social information during the activity, enhancing their attention to the external world and reducing the occurrence of stereotypical behaviors. (2) Motor imitation learning. In team sports, children with ASD may adjust their own behavior by observing the actions of others, improving their movement flexibility and thus reducing dependence on fixed stereotypical behaviors.

### 4.3 Analysis of sources of heterogeneity

The heterogeneity in existing studies may affect the results of the meta-analysis. Although this study integrated multiple RCT studies through meta-analysis, and the results indicate that the overall heterogeneity, as well as that in subgroup analyses, is relatively low. However, due to significant differences in intervention methods, sample characteristics, and measurement tools among the studies, some degree of heterogeneity may still exist. For instance, the diversity in intervention methods: the types of exercise, intensity, frequency, and setting (indoor vs. outdoor) used in different studies may vary considerably, which could affect the comparability of the results. Differences among individuals with ASD: the participants in different studies varied in terms of age, symptom severity, and the prevalence of comorbidities (such as ADHD), which could influence the stability of intervention effects. Although we controlled for some influencing factors in the subgroup analyses, the impact of heterogeneity could not be completely eliminated. Future research should further standardize experimental designs to reduce variability between studies.

Notably, gender emerged as a critical heterogeneity factor. Our cohort exhibited a significant male predominance (male-to-female ratio 3.5:1), aligning with the established male-biased prevalence in autism spectrum disorder (ASD) (Lai and Szatmari, 2020). This imbalance may mechanistically contribute to intervention heterogeneity: testosterone in males may exacerbate restricted/repetitive behaviors via dopaminergic pathways, whereas estrogen-mediated neuroprotection in females could modulate sensory processing (Baron-Cohen et al., 2015). Ball sports requiring rapid motor adjustments may preferentially engage dopaminergic systems, potentially explaining their pronounced efficacy in male-dominated cohorts. Concurrently, females with ASD often exhibit masked stereotypies due to social camouflaging (Keluskar et al., 2021), which may introduce measurement bias in mixed-gender intervention assessments. Furthermore, social rewards embedded in group activities (e.g., basketball) may differentially reinforce structured exercise adherence in males, given their heightened responsiveness to rule-based reinforcement systems (Tseng et al., 2016).

### 4.4 Research strengths and limitations

#### 4.4.1 Main strengths of the study

(1) Use of meta-analysis to enhance the evidence level of the study. By using meta-analysis to synthesize multiple studies, this approach reduces individual study biases compared to single experimental studies, improving the external validity of the results. Additionally, we employed strict inclusion and exclusion criteria to ensure high-quality data, providing stronger evidence for the impact of physical interventions on stereotyped behaviors in ASD. (2) Refining key variables in physical exercise to improve the interpretability of the results. This study not only validated the overall impact of physical exercise on stereotyped behaviors but also conducted subgroup analyses on key factors such as exercise type, intervention duration, frequency, session length, and participation format. This provides a more refined basis for selecting different forms of exercise. It also offers more practically relevant recommendations for developing personalized intervention programs. (3) Exploring the pathways through which physical exercise affects stereotyped behaviors in relation to neural mechanisms. This study not only focused on intervention effects but also integrated existing neuroscience research to explore how physical exercise might reduce stereotyped behaviors by enhancing vestibular perception, proprioception, sensory integration, and social cognition. Compared to studies that focus solely on behavioral outcomes, our research takes a deeper theoretical approach. (4) The results have practical implications for clinical rehabilitation and educational practice. Our findings provide practical guidance for fields such as special education, rehabilitation training, and family intervention. For example, the results indicate that group exercises are more effective than individual exercises. This suggests that rehabilitation training should incorporate more group sports programs, such as basketball or soccer, rather than focusing solely on running or swimming. Intervention programs lasting more than 60 min are more effective, offering practical time guidelines for practitioners.

#### 4.4.2 Limitations of the study

(1) The heterogeneity of existing studies may affect the results of the meta-analysis. Although this study integrated multiple RCTs through meta-analysis, the significant differences in intervention methods, sample characteristics, measurement tools, and other factors across studies may lead to a degree of heterogeneity. (2) Lack of long-term follow-up data makes it difficult to assess the long-term effects of the intervention. The majority of RCTs included in this study were short-term interventions (8–12 weeks), and few studies followed up on the effects after 6 months, 1 year, or even longer. Therefore, we are unable to determine whether the effect of physical exercise in reducing stereotyped behaviors is sustainable over the long term. Future research should adopt longitudinal study designs to track the stereotyped behaviors of ASD individuals over time to clarify the sustained effects of physical exercise. (3) The dose–response relationship of physical exercise still requires further investigation. This study found a certain dose–response relationship between the frequency and duration of physical exercise and intervention outcomes, but it did not define the optimal “dose” (i.e., the most suitable combination of frequency, duration, and intensity of exercise for ASD children). For example, we found that 45–60 min of exercise intervention was ineffective, while >60 min was effective, but this result still requires further validation. Future studies could adopt dose-effect experimental designs to explore the optimal combination of exercise intensity, duration, and frequency for reducing stereotyped behaviors. Furthermore, the randomized controlled trials included in this study failed to adequately report exercise intensity parameters, which complicates the analysis of dose–response relationships. Future research should prioritize standardized reporting of intensity metrics (e.g., heart rate zones, MET values, or Borg scale ratings) as a critical moderating variable. (4) Reliance on behavioral measurements with a lack of support from neurophysiological indicators. The measurement of stereotyped behaviors in this study mainly relied on behavioral assessment scales (e.g., the Repetitive Behavior Scale-Revised, RBS-R), rather than directly measuring the effects of exercise on brain function, neural networks, or neurotransmitter levels. Future research could integrate neuroscience technologies such as functional magnetic resonance imaging (fMRI), electroencephalography (EEG), and eye-tracking to further verify the neural mechanisms through which physical exercise reduces stereotyped behaviors, thus improving the physiological interpretability of the study. (5) The impact of exercise on different subtypes of ASD individuals still requires further exploration. ASD is highly heterogeneous, and different subtypes (e.g., high-functioning vs. low-functioning ASD, comorbid anxiety/ADHD vs. pure ASD) may respond differently to physical interventions. Most current studies have not conducted stratified analysis based on ASD subtypes, so our results may not be applicable to all ASD individuals. Future studies should use more refined stratified analysis methods to explore the response patterns of ASD individuals with different characteristics to physical exercise.

#### 4.4.3 Summary and future outlook

This study systematically evaluated the effects of physical exercise on the improvement of stereotyped behaviors in children with ASD and revealed the impact of different types of exercise, intervention duration, frequency, and timing. These findings not only provide empirical support for physical intervention programs for children with ASD but also guide future research directions. However, the study still has some limitations, such as significant heterogeneity between studies, lack of long-term follow-up data, and insufficient exploration of the neural mechanisms. Therefore, future research should: (1) further standardize physical intervention programs to reduce heterogeneity across studies. (2) Adopt longitudinal study designs to explore the long-term sustainability of intervention effects. (3) Combine neuroimaging and neurophysiological methods to explore the neural mechanisms through which physical exercise impacts stereotyped behaviors in children with ASD. (4) Study the differential responses to physical interventions in individuals with different ASD subtypes to develop more targeted intervention strategies.

## 5 Conclusion

This study systematically evaluated the effects of physical exercise on restricted and repetitive behaviors (RRBs) in children with autism spectrum disorder (ASD) based on meta-analysis. It also further explored the impact of exercise type, intervention duration, frequency, session time, participation format, and the participants’ age on intervention outcomes through subgroup analysis. The results indicate that different types of exercise and intervention parameters have significant differences in their effectiveness in improving stereotyped behaviors, suggesting that the mechanisms underlying physical exercise may be jointly regulated by various factors.
